# Solvent-free, visible-light photocatalytic alcohol oxidations applying an organic photocatalyst

**DOI:** 10.3762/bjoc.12.229

**Published:** 2016-11-09

**Authors:** Martin Obst, Burkhard König

**Affiliations:** 1 Institute of Organic Chemistry, University of Regensburg, Universitätsstraße 31, 93040 Regensburg, Germany

**Keywords:** benzylic alcohol, oxidation, photocatalysis, solvent free, visible light

## Abstract

A method for the solvent-free photocatalytic conversion of solid and liquid substrates was developed, using a novel rod mill apparatus. In this setup, thin liquid films are realized which is crucial for an effective photocatalytic conversion due to the low penetration depth of light in heterogeneous systems. Several benzylic alcohols were oxidized with riboflavin tetraacetate as photocatalyst under blue light irradiation of the reaction mixture. The corresponding carbonyl compounds were obtained in moderate to good yields.

## Introduction

According to a classification made by Wilhelm Ostwald, one of the pioneers in the field of physical chemistry and Nobel Prize laureate 1909, chemistry can be divided into the four sub-disciplines thermochemistry, electrochemistry, photochemistry, and mechanochemistry, depending on the kind of energy involved in a chemical process [1]. Among these, mechanochemistry is nowadays a well-established method for the solvent-free conversion of solid reactants. The reaction is driven by mechanical energy, which is for instance realized by grinding in ball mills or pestle and mortar. Obviously, this method possesses several advantages compared to reactions, which are performed in solution: toxic solvents and wastes are avoided, making the process more environmentally friendly and sustainable. Furthermore, quantitative yields can be achieved, avoiding work-up and laborious purification [2]. Moreover, solubility problems, like insolubility of one reactant or the different polarity of two reactants, are solved. Examples for mechanochemical syntheses include stoichiometric reactions such as the Knoevenagel condensation and the Wittig reaction, but also reactions catalyzed by metal catalysts, like the Sonogashira coupling and the Suzuki coupling [1].

Photocatalysis, as a part of Ostwald’s sub-discipline photochemistry, is a rather new field of great academic interest. Namely, visible-light photocatalysis applying an organic, redox-active catalyst allows mild and efficient transformations. By exciting the photocatalyst, which then exchanges electrons with the substrate, light energy is converted into chemical energy [3]. This approach avoids expensive reagents and harsh reaction conditions, which are improvements compared to conventional processes. Considering that, we envisaged combining photocatalysis with mechanochemistry, thereby making use of the advantages of both disciplines. In such an approach, solid substrates would be grinded under visible light irradiation. In contrast to mechanochemistry, the process would be driven by light energy and not by mechanical energy, but profit from the absence of toxic solvents, high concentrations of the substrate, and easy work-up. Furthermore, undesired effects of the solvent like hydrogen-atom transfer or the formation of byproducts could be excluded.

For liquid substrates, some examples for photocatalytic, solvent-free conversions are reported, such as the oxidation of benzyl alcohol to benzaldehyde [4] and the oxidation of benzenes to phenols [5]. Another field of solvent-free photocatalysis is the application of heterogeneous, semiconducting photocatalysts, often based on titanium dioxide and other metal oxides [6–8]. However, to the best of our knowledge, no solvent-free visible-light driven transformation of a solid substrate applying an organic photocatalyst has been reported yet.

In this work, we present a novel milling apparatus, which we developed especially for the conversion of solid substrates. Applying this apparatus, the solvent-free oxidation of various benzylic alcohols to their corresponding carbonyl compounds using riboflavin tetraacetate as photocatalyst under blue light irradiation was performed. Furthermore, the oxidation of a liquid benzylic alcohol is presented as well.

## Results and Discussion

For our investigations on solvent-free photocatalytic conversions, we chose riboflavin tetraacetate (RFTA) as photocatalyst. RFTA and flavin derivatives in general are well-known blue-light absorbing photocatalysts, which can oxidize a variety of substrates under aerobic conditions with oxygen as terminal oxidant. Flavins were studied extensively for the oxidation of alcohols, amines, methylbenzenes, styrenes, and phenylacetic acids [9–12]. Some derivatives were also immobilized on silica gel and applied in the oxidation of benzyl alcohols [13]. Furthermore, it was shown that the oxidation power of RFTA can be increased by coordination to scandium triflate [14]. Recently, the *E*/*Z*-isomerization of olefins with riboflavin as catalyst was reported [15], which was also used for cyclization to form coumarins [16].

In this work, we focus on benzylic alcohols as substrates since their oxidation to the corresponding carbonyl compounds can be simply performed in acetonitrile/water mixtures. Thus, they are suitable model substrates for the investigation of our solvent-free principle.

**Figure 1:**
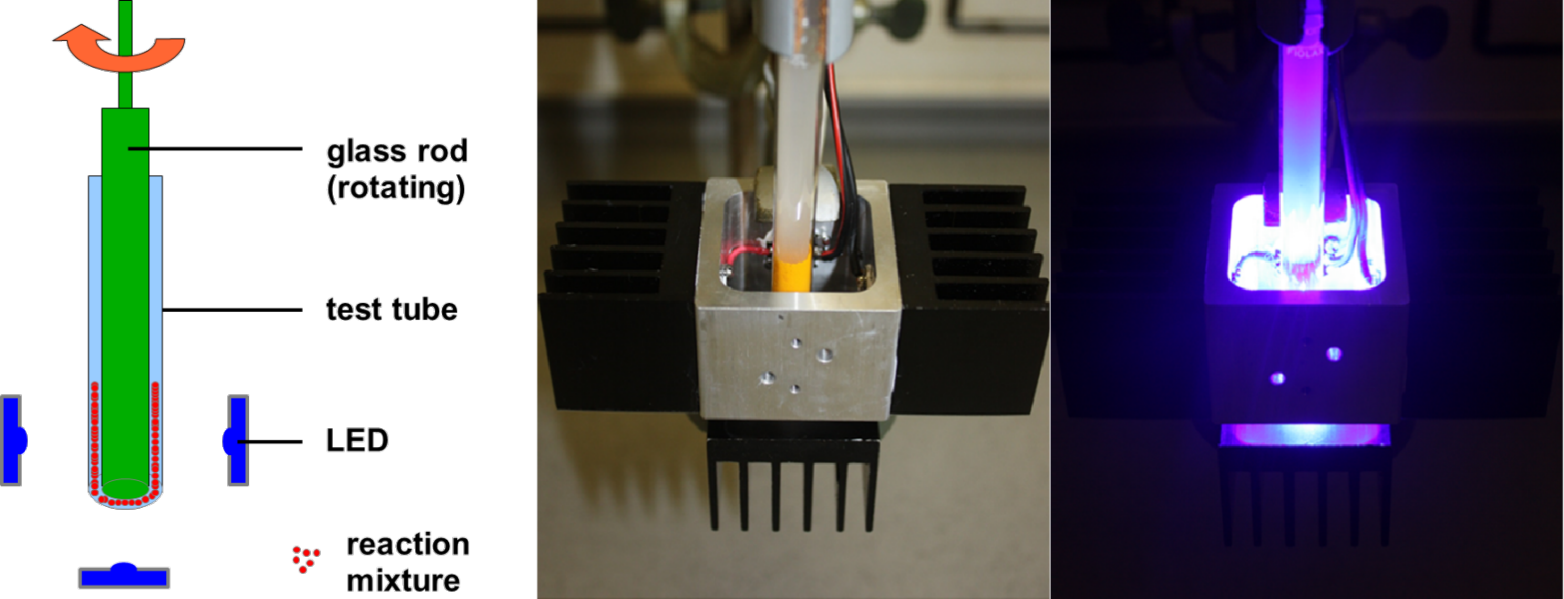
Rod mill, schematic (left) and photographs (middle and right).

In mechanochemistry, reactions are performed by grinding the reaction mixture in ball mills, such as the vibrational ball mill or the planetary ball mill, or with a pestle and mortar. In preliminary studies, we tested a vibrational ball mill for photocatalytic oxidations with riboflavin tetraacetate, using a transparent milling chamber. However, this reactor proved to be unsuitable: the solid reaction mixture prevented light to reach the photocatalyst, both by attachment of small amounts of solid to the inner side and shielding within the milling chamber. The low penetration depth of light led us to the conclusion that thin interfaces of reactants, combined with continuous grinding, are necessary. Based on these considerations, we constructed a rod mill, consisting of a test tube which contains the reaction mixture, a glass rod, which is fixed to a stirrer, and an LED frame with 5 LEDs (λ = 455 nm). Figure 1 shows a schematic representation and photographs. Before reaction, the solid substrate and photocatalyst are gently homogenized with a spatula, followed by grinding with pestle and mortar. The reaction mixture is then filled into the test tube. Subsequently, the rotating glass rod is pressed into the test tube, which leads to vertical migration of the reaction mixture and the formation of a film between the inner wall of the test tube and the glass rod. The reaction is carried out by rotation and irradiation with the LEDs from the outside.

**Scheme 1:**
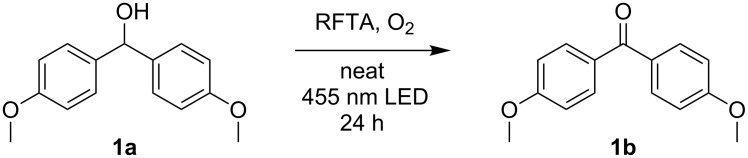
Oxidation of 4,4’-dimethoxybenzhydrol (**1a**) to 4,4’-dimethoxybenzophenone (**1b**).

We started our investigations with the oxidation of 4,4’-dimethoxybenzhydrol (**1a**) to 4,4’-dimethoxybenzophenone (**1b**), applying 10% RFTA (Scheme 1).

In this initial trial, the product could be obtained in 72% yield. Subsequently, we varied the amount of RFTA (Table 1).

**Table 1:** Conditions and yields for the oxidation of 4,4’-dimethoxy-benzhydrol (**1a**).

Entry	RFTA (%)	Yield (%)

1	10	72
2	5	74
3	2	69
4	1	61
5^a^	10	0
6	–	traces
7^b^	5	traces
8^c^	5	traces
9^d^	5	78

^a^In the dark; ^b^in the dark at 85 °C; ^c^with cooling; ^d^no rotation.

Lowering the catalyst concentration from 10% to 5% does not significantly influence the yield (entry 2, Table 1, yield in average of four trials). Even a further decrease to 2% (entry 3, Table 1) causes no essential decline. However, at a RFTA concentration of 1% (entry 4, Table 1), the isolated yield was only 61%; very low catalyst concentrations are problematic due to inactivation of a certain portion of the catalyst, which might be due to burning.

No product was formed in the dark (entry 5, Table 1) and only traces were obtained without photocatalyst (entry 6, Table 1). However, in this control reaction, melting of the substrate was observed. All reaction mixtures containing RFTA appeared solid. The fact that the substrate was molten without RFTA leads to the conclusion that the blue light irradiation was heating the substrate and temperatures higher than its melting point (67–70 °C) occurred. The temperatures of the reaction mixtures were measured using an IR thermometer. Though, values not higher than 42 °C were obtained. This could be explained by the fact that the IR thermometer records only the surface temperature of the test tube; the temperature within the reaction mixture might be above this value. When the reaction mixture was heated to 85 °C in the dark, melting was observed (entry 7, Table 1) and only traces of product were formed, showing that the oxidation of the substrate is not only due to heating. For further investigation, the reaction was performed within a continuous water cooling (entry 8, Table 1). In this setup, no product was formed. Therefore, the heating effect of the blue light was found to be necessary to perform the reaction: melting of the substrate leads to mobility of the substrate and catalyst molecules, ensuring the occurrence of the catalytic cycle. When the rotation was switched off after the formation of the solid film (2 min), followed by LED irradiation (entry 9, Table 1), the product was obtained in similar yield compared to the reactions performed under grinding (entry 2, Table 1), indicating that the grinding does not contribute to the essential heating. Hence, light irradiation fulfils a double function: the excitation of the photocatalyst and melting of the substrate, inducing mobility of the molecules. As mentioned above, the reaction mixtures containing RFTA appeared solid. Possibly, small droplets of molten substrate were formed; due to the presence of RFTA, no homogeneous liquid phase was observed. In mechanochemistry, the formation of hot spots by friction heating with high local temperatures and the existence of liquid eutectic states are believed to be responsible for chemical transformations [1]. In contrast, in our rod mill apparatus, heating is not induced by mechanical but by electromagnetic energy input, realized by blue light irradiation. Furthermore, the heat input is not the primary cause for the reaction, but its prerequisite.

**Scheme 2:**
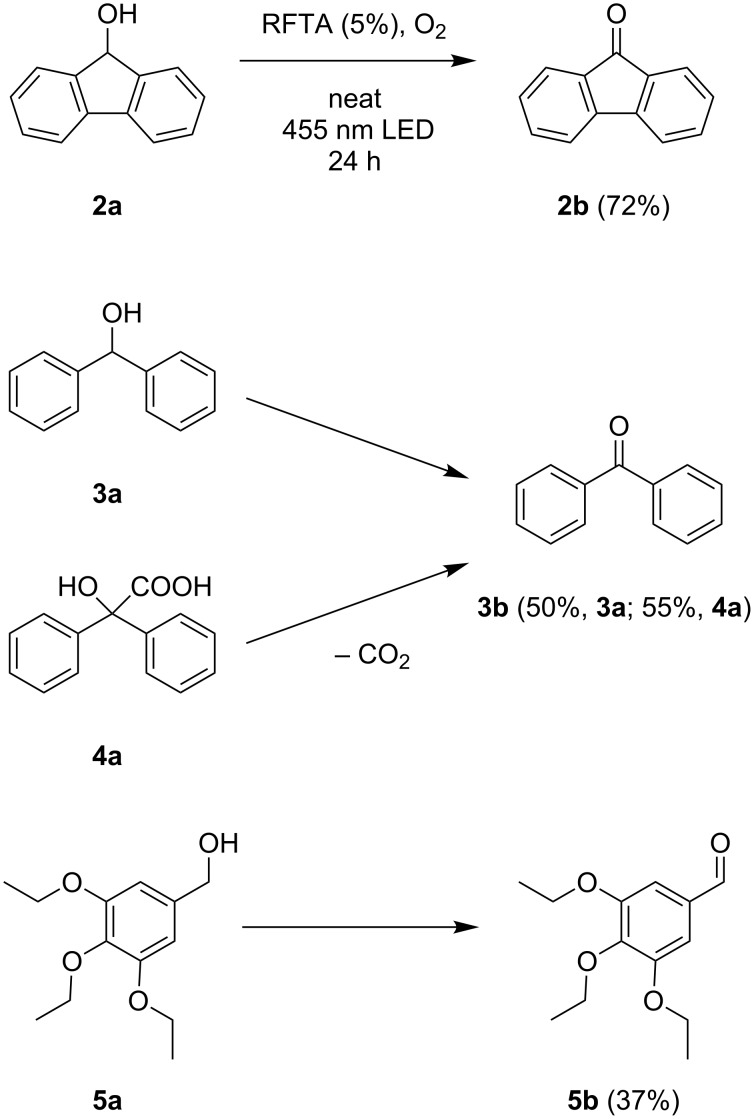
Scope for benzylic alcohol oxidation and obtained yields.

Subsequently, the scope of the benzylic alcohol oxidation was investigated, applying 5% of RFTA. Four solid alcohols were oxidized to their corresponding ketones or aldehydes, including fluorenol (9-hydroxyfluoren), benzhydrol, benzilic acid, and 3,4,5-triethoxybenzyl alcohol (**2a–5a**). Scheme 2 shows the products and isolated yields, which are in the range from 37% to 72%. In contrast to 4,4’-dimethoxybenzhydrol, a liquid paste was observed in all cases, indicating melting of the substrate or the product, which maintains mobility. The oxidation of benzilic acid and benzhydrol was also performed using an LED setup containing only 4 LEDs instead of 5 LEDs like in the frame rod mill. Here, the obtained yields were significantly lower (36% and 30% vs 55% and 50%). This shows that the amount of energy reaching the reaction mixture plays a crucial role.

**Scheme 3:**
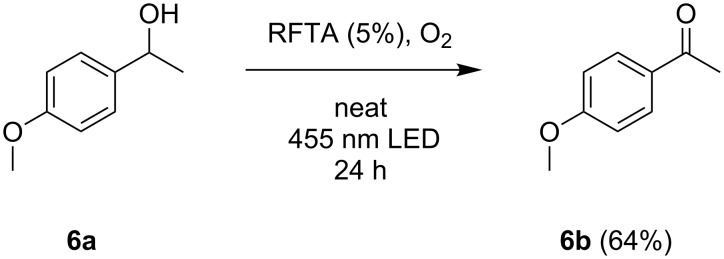
Oxidation of 4-methoxyphenyl methyl carbinol (**6a**) to 4-methoxyacetophenone (**6b**).

Furthermore, the suitability of the rod mill for the conversion of liquid substrates was tested. 4-Methoxyphenyl methyl carbinol was oxidized applying 5% of RFTA (Scheme 3).

The crude ^1^H NMR spectrum recorded after the reaction shows a quantitative conversion of the staring material and no visible side product formation (see Experimental). In general terms, this shows the potential of the method for an efficient synthesis with direct product formation, facilitating work-up. However, the isolated yield was only 64% (average of two trials), indicating loss in the column purification process and decomposition of a part of the product in the reaction mixture.

## Conclusion

We have developed a method for the solvent-free photocatalytic conversion of solid and liquid substrates using a novel rod mill apparatus. The applicability of the rod mill was shown for the oxidation of benzylic alcohols with riboflavin tetraacetate as photocatalyst under blue light irradiation; the products were isolated in moderate to good yields. In case of the solid benzyl alcohols, the reactions were found to proceed via the molten state of the substrates or products, which enables mobility of the substrate and catalyst molecules and the occurrence of the catalytic cycle. Thus, light fulfils the double function of both the excitation of the photocatalyst and heating of the reaction mixture.

To summarize, the new combination of photocatalysis and mechanochemistry, realized in a simple rod mill apparatus is an alternative to conventional reaction setups. It may provide advantages for the conversion of starting materials or catalysts with no or low solubility in the same solvent and reactions benefitting from high substrate concentrations.

## Experimental

### Materials and methods

NMR spectra were recorded on a Bruker Avance 300. 4,4’-Dimethoxybenzhydrol was purchased from Alfa Aesar, 9-hydroxyfluoren, benzhydrol, and 3,4,5-triethoxybenzaldehyde from Sigma-Aldrich, benzilic acid from Merck Schuchardt and 4-methoxyphenyl methyl carbinol from Fluorochem. Riboflavin tetraacetate was synthesized from riboflavin (Acros Organics) according to a procedure reported in the literature [17].

Technical data of the rod mill apparatus: 4 LEDs (455 nm, 700 mA) on a square aluminium frame (inner diameter 35 mm) and one LED below the test tube were used. The distance of each LED to the test tube was 10 mm. The diameter of the glass rod was 8 mm and the inner diameter of the test tube was 9 mm.

### Synthesis

General procedure: The substrate and riboflavin tetraacetate were weighed into a mortar in stoichiometric amounts and gently homogenized with a spatula, followed by grinding with a pestle and mortar. The reaction mixture was filled into the rod mill test tube and the mass was determined. Subsequently, the rotating glass rod was pressed into the test tube, forming a film between the test tube and the glass rod. The reaction was carried out by rotation of the glass rod (80 rpm) and parallel irradiation with the 455 nm LEDs for 24 hours. After the reaction, the product was isolated by flash column chromatography (gradient of ethyl acetate in petroleum ether).

#### 4,4’-Dimethoxybenzophenone (**1b**)


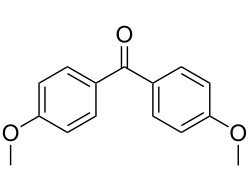


^1^H NMR (300 MHz, CDCl_3_) 7.79 (m, 4H, CH), 6.96 (m, 4H, CH), 3.88 (s, 6H, CH_3_); EIMS *m*/*z*: 242.0924 [M]^+^.

**Table 2:** Amounts of 4,4’-dimethoxybenzhydrol (**1a**) and riboflavin tetraacetate (RFTA) and yields for the oxidation to 4,4’-dimethoxybenzo-phenone (**1b**).

Entry^a^	Amount, weighed (mg/mmol)		(trial)	Total mass for reaction (mg)	Yield (%)

	**1a**	RFTA			

1	85.5/0.35	19.1/0.035	–	77.2	72
2	85.5/0.35	9.5/0.018	1	76.7	70
			2	76.3	79
			3	81.4	70
			4	83.2	76
3	97.7/0.4	4.4/0.008	–	83.5	69
4	97.7/0.4	2.2/0.004	–	76.6	61
9	85.5/0.35	9.5/0.018	–	81.9	78

^a^In Table 1.

#### 9-Fluorenone (**2b**)


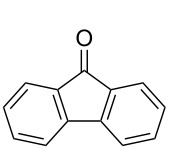


9-Hydroxyfluorene (82.0 mg, 0.45 mmol) and RFTA (12.3 mg, 0.023 mmol) were weighed and 70.6 mg of the mixture was used for reaction. Yield: 72%. ^1^H NMR (300 MHz, CDCl_3_) 7.66 (d, 2H, CH), 7.54–7.47 (m, 4H, CH), 7.30 (m, 2H, CH); EIMS *m*/*z*: 180.0579 [M]^+^.

#### Benzophenone (**3b**)


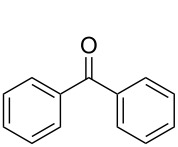


**3b** from **3a**: Diphenylmethanol (73.3 mg, 0.40 mmol) and RFTA (10.9 mg, 0.020 mmol) were weighed and 74.0 mg of the mixture was used for reaction. Yield: 50%. ^1^H NMR (300 MHz, CDCl_3_) 7.83–7.79 (m, 2H, CH), 7.62–7.57 (m, 2H, CH), 7.51–7.46 (m, 4H, CH); EIMS *m*/*z*: 182.0730 [M]^+^.

**3b** from **4a**: Benzylic acid (91.3 mg, 0.40 mmol) and RFTA (10.9 mg, 0.020 mmol) were weighed and 89.2 mg of the mixture was used for reaction. Yield: 55%.

#### 3,4,5-Triethoxybenzaldehyde (**5b**)


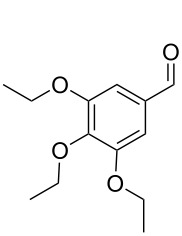


3,4,5-Triethoxybenzyl alcohol (96.1 mg, 0.40 mmol) and RFTA (10.9 mg, 0.020 mmol) were weighed and 82.3 mg of the mixture was used for reaction. Yield: 37%. ^1^H NMR (300 MHz, CDCl_3_) 9.82 (s, 1H, CHO), 7.08 (s, 1H, CH), 4.17–4.09 (m, 6H, CH_2_), 1.45 (t, 6H, CH_3_), 1.36 (t, 3H, CH_3_).

#### 4-Methoxyacetophenone (**6b**)


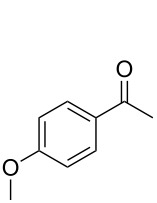


**Figure 2:**
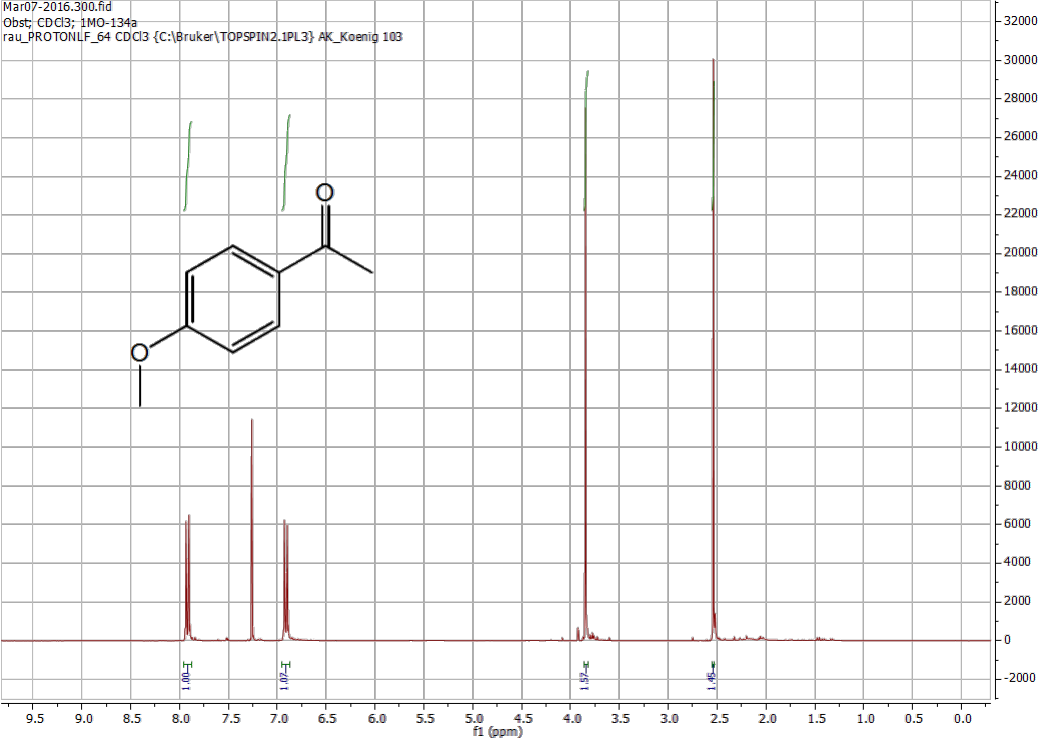
^1^H NMR (crude) of 4-methoxyacetophenone **6b**.

Riboflavin tetraacetate was grinded with pestle and mortar before use. 4-Methoxyphenyl methyl carbinol and riboflavin tetraacetate were weighed into the rod mill test tube and the mixture was sonicated for 1 min. The rotating glass rod was pressed into the test tube, forming a film between the test tube and the glass rod. The reaction was carried out by rotation of the glass rod (80 rpm) and parallel irradiation with the 455 nm LEDs for 24 hours. After the reaction, the product was isolated by flash column chromatography (gradient of ethyl acetate in petroleum ether). **Trial 1:** 4-Methoxyphenyl methyl carbinol (82.2 mg, 0.54 mmol) and RFTA (14.0 mg, 0.03 mmol) were used. Yield: 63%. **Trial 2:** 4-Methoxyphenyl methyl carbinol (178.5 mg, 1.17 mmol) and RFTA (31.9 mg, 0.06 mmol) were used. Yield: 65%. ^1^H NMR (300 MHz, CDCl_3_, Figure 2) 7.94 (m, 2H, CH), 6.93 (m, 2H, CH), 3.87 (s, 3H, OCH_3_), 2.55 (s, 3H, CH_3_); CIMS: 151.0771 [M + H]^+^.

## Supporting Information

File 1 Video showing the rod mill apparatus and the procedure of loading and reaction.

## References

[1] James S L, Adams C J, Bolm C, Braga D, Collier P, Friščić T, Grepioni F, Harris K D M, Hyett G, Jones W (2012). Chem Soc Rev.

[2] Trotzki R, Hoffmann M M, Ondruschka B (2008). Green Chem.

[3] Reckenthaeler M, Griesbeck A G (2013). Adv Synth Catal.

[4] Ohkubo K, Suga K, Fukuzumi S (2006). Chem Commun.

[5] Ohkubo K, Hirose K, Fukuzumi S (2015). Chem – Eur J.

[6] Feng W, Wu G, Li L, Guan N (2011). Green Chem.

[7] Yuan R, Fan S, Zhou H, Ding Z, Lin S, Li Z, Zhang Z, Xu C, Wu L, Wang X (2013). Angew Chem, Int Ed.

[8] Lang X, Chen X, Zhao J (2014). Chem Soc Rev.

[9] Cibulka R, Vasold R, Koenig B (2004). Chem – Eur J.

[10] Svoboda J, Schmaderer H, Koenig B (2008). Chem – Eur J.

[11] Lechner R, Koenig B (2010). Synthesis.

[12] Lechner R, Kuemmel S, Koenig B (2010). Photochem Photobiol Sci.

[13] Schmaderer H, Hilgers P, Lechner R, Koenig B (2009). Adv Synth Catal.

[14] Muehldorf B, Wolf R (2015). Chem Commun.

[15] Metternich J B, Gilmour R (2015). J Am Chem Soc.

[16] Metternich J B, Gilmour R (2016). J Am Chem Soc.

[17] Larson R A, Stackhouse P L, Crowley T O (1992). Environ Sci Technol.

